# Analysis of Heterogeneous Gelation Dynamics and Their Application to Blood Coagulation

**DOI:** 10.3390/gels4030059

**Published:** 2018-07-09

**Authors:** Toshiaki Dobashi, Takao Yamamoto

**Affiliations:** 1 Division of Molecular Science, Graduate School of Science and Technology, Gunma University, Kiryu, Gunma 376-8515, Japan; 2 Division of Pure and Applied Science, Graduate School of Science and Technology, Gunma University, Kiryu, Gunma 376-8515, Japan; tyam@gunma-u.ac.jp

**Keywords:** heterogeneous gelation dynamics, moving boundary picture, phase transition dynamics, kinetic coefficient, blood coagulation

## Abstract

We present a scaling model based on a moving boundary picture to describe heterogeneous gelation dynamics. The dynamics of gelation induced by different gelation mechanisms is expressed by the scaled equation for the time taken for development of the gel layer with a few kinetic coefficients characterizing the system. The physical meaning obtained by the analysis for a simple boundary condition from the standpoint of the phase transition shows that the time development of the gelation layer depends on whether the dynamics of the order parameter expressing the gelation of the polymer solution is fast or slow compared with the diffusion of the gelators in the heterogeneous gelation. The analytical method is used to understand the coagulation of blood from various animals. An experiment using systems with plasma coagulation occurring at interfaces with calcium chloride solution and with packed erythrocytes is performed to provide the data for model fitting and it is clarified that a few key kinetic coefficients in plasma coagulation can be estimated from the analysis of gelation dynamics.

## 1. Introduction

Commonly used gels are prepared using chemical reagents while mixing, by lowering the temperature and by the irradiation of high-energy electromagnetic waves, such as an electron beam and UV, where the cross-linking sites of polymer networks are dispersed randomly in gels, resulting in a macroscopically isotropic and homogeneous structure. This is because the maximum entropy of easily deformed polymer segments between cross-linking sites is obtained when the number of conformational states is maximum. Therefore, to prepare anisotropic gels some asymmetry, such as an asymmetric external force on the gel, should be required. To date, most studies performed on gelation have been on homogeneous systems [1,2,3]. In industry, however, gels are frequently prepared at an interface between two phases. Following the classification of chemical reactions into homogeneous and heterogeneous reactions, we refer to the gelation occurring in a homogeneous phase as homogeneous gelation and the gelation involving reactions at interfaces between different phases as heterogeneous gelation. In 1954, Thiele reported the fabrication of anisotropic beads by dripping an alginate solution in a bath of calcium chloride aqueous solution [4]. Thiele and coworkers prepared anisotropic channel-like alginate gels induced by diffusion of various multivalent cations [5]. These are among the first examples of heterogeneous gelation, on which many studies have now been reported [6,7,8,9,10,11,12,13,14,15,16,17]. The most important gels yielded by heterogeneous gelation are gels in parts of the human body. They are anisotropic, with the anisotropy often having a gradient, such as blood vessel walls [18]. Although the mechanism of biosynthesis is rather complex, most biotissues are yielded from a surface or a point and the growth is directional [19]. Therefore, understanding heterogeneous gelation is important from both industrial and biological viewpoints. 

Among the systems that undergo heterogeneous gelation, polymer solutions in contact with solutions containing gelators such as cross-linkers have many promising applications. As a typical example of an anisotropic gel whose structure has been studied is a cylindrical gel prepared by the dialysis of sodium alginate aqueous solution in calcium chloride aqueous solution [4,5,6,7,8,9,10,20]. Maki et al., performed a small-angle X-ray scattering study on the structure of the alginate gel and showed that the polymer orientation is perpendicular to the flow of the gelators [20]. They showed that the gel is highly anisotropic near the surface (dialysis tube) but becomes isotropic toward the center. It has been shown for many combinations of polymers and gelators that the orientation is always similar to that observed for the alginate [14,15,21]. By changing the boundary conditions, we can prepare anisotropic gels with various shapes such as spherical gels and fiber-like gels [8]. The birefringence distribution of the gels is consistent with the orientation of polymer molecules perpendicular to the flow of the gelators [8]. In these systems, polymer molecules cannot permeate into the gel and the gelators selectively diffuse into the already yielded gel, resulting in the cross-linking of polymers at the reaction front. Since the polymers should have a high affinity with the gelators, they are in contact with the interface over a large contact area, which results in molecular orientation. Study of the microrheological events occurring on polymer molecules in the boundary layer at the sol-gel interface is challenging, although such studies should be performed in the near future. 

For applications, the present approach to yielding anisotropy has an advantage of gelation in a self-organized manner under mild conditions. Several elaborate gels having unique properties have been developed. Gong’s group at Hokkaido University used a double network gelation involving the contact of synthetic polymer solutions with gelator solutions to prepare a variety of tough anisotropic artificial substitutes such as artificial cartilage [22,23]. Furusawa et al. prepared epithelial lumen-like engineered tissues by the contact of collagen solutions with buffer solutions having a high pH due to spinodal decomposition [24]. Konno et al., prepared a multilayer cylindrical gel consisting of an inner isotropic gel layer and an outer highly anisotropic gel layer [25,26]. The release of drugs from gel particles having such multilayers may exhibit different behavior from the common exponential behavior observed for homogeneous particles and used for stepwise or constant drug release, similarly to capsules or spheres having layer-by-layer wall membranes [27]. 

On the other hand, the results of studies on the dynamics of heterogeneous gelation have not been organized and almost no applications of such gelation dynamics have been reported, although basic data on the gelation dynamics for simple polymer systems that undergo gelation in contact with gelator solutions have been accumulated [28,29,30,31,32,33]. Measurable physical quantities characterizing the gelation process are the gel fraction and the molecular weight distribution in the sol fraction in homogeneous gelation [1], whereas they are the gel volume and the degree of orientation of the gel in heterogeneous gelation [8]. In most cases, gel layers are formed at the interface between the polymer solution and the gelator solution and extended into the polymer solution while keeping the sol-gel interface clear [8]. Yamamoto et al., developed an irreversible thermodynamic theory focusing on the motion of the sol-gel boundary (moving boundary (MB) picture) [28,29]. 

In this article, we attempt to reorganize the experimental results on the basis of the MB picture, focusing on the role of the gelator. Then we propose a way of classifying the previously reported systems into several types as a first step toward understanding the characteristics of heterogeneous gelation dynamics. The gelation dynamics are expressed using universal scaling equations corresponding to the gelation mechanism, such as gelation induced by the inflow of cross-linkers (Case 1), by solvent exchange (Case 2), by the inflow of catalysts (Case 3), by the exchange of solutes having very different diffusion constants (Case 4) and by nucleation at low supersaturation (Case 5) (see Section 2.3 for details). In Section 3, we discuss the physical meaning of isotropic and anisotropic gelation from the standpoint of phase separation. By fitting data for gelation dynamics whose gelation mechanism is unknown to the universal scaling equation for Cases 1–5, we can identify the type of the gelation of the system. Furthermore, we can determine kinetic coefficients from the fitting parameters. The second aim of this article is to apply the analytical method to one of the most important biomedical processes, blood coagulation. Although some properties of the blood of patients can be obtained from conventional biochemical tests and the time required for blood coagulation, we have few means of estimating kinetic properties in blood coagulation, which is part of the missing link between blood properties and disorder in blood coagulation. Analysis of the dynamics of blood coagulation by fitting the data to the universal scaling equation enables us to extract information on kinetic coefficients in the process that cannot be obtained by static measurements. In Section 4, we describe the application of the analytical method for gelation dynamics to two model systems of blood coagulation to determine several key kinetic coefficients relating to blood coagulation.

## 2. Experimental Results on Gelation Dynamics and Classification of Systems by Moving Boundary Picture

We propose a generalized model of heterogeneous gelation and show the observed gelation dynamics of chitosan solution [30] as a typical example in Section 2.1. Then we demonstrate a theoretical analysis of the dynamics based on the MB picture in Section 2.2. In Section 2.3 we discuss the factors which determine the characteristics of the dynamics in Section 2.2 and propose a way of classifying of various systems according to the key factors.

### 2.1. Gelation Dynamics of Chitosan Solution in Contact with Solutions with High pH

**Figure 1:**
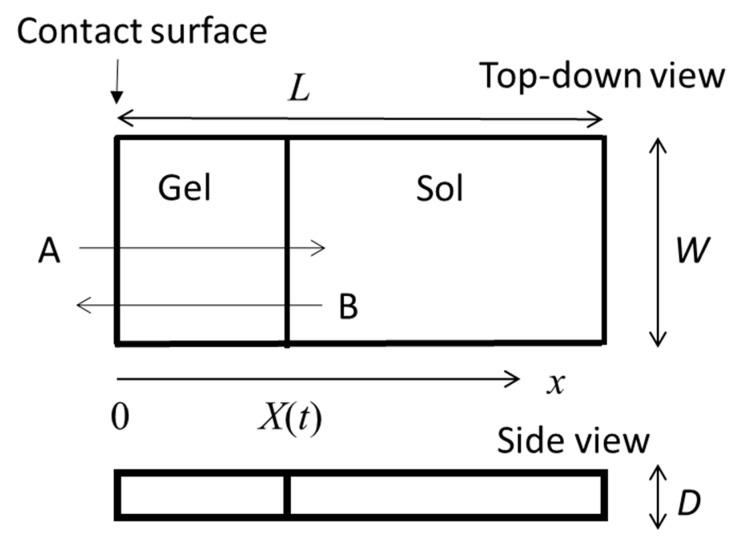
Illustration of one-dimensional gel layer growth from the contact surface between polymer solution enclosed in an L (length) × W (width) × D (depth) rectangular cell in an immersing gelator solution (left-hand side of the cell) induced by inflow of A and outflow of B. The x-axis is chosen to be perpendicular to the contact surface and is oriented in the direction from the immersing gelator solution to the polymer solution. The origin of the x-axis is chosen at the contact interface. *X*(*t*) denotes the gel layer thickness at immersion time *t*. In the gelation of chitosan solution, the gelator solution and polymer solution are NaOH aqueous solution and chitosan in acetic acid aqueous solution, respectively.

**Figure 2:**
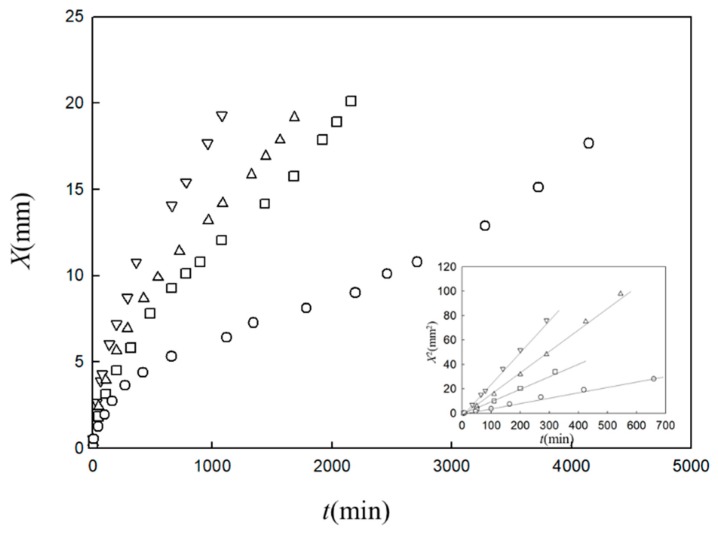
Time course of gel thickness *X* observed for one-dimensional growth of chitosan gel induced in 2 wt. % chitosan in 2 wt. % acetic acid aqueous solution in contact with sodium hydroxide aqueous solution with concentrations of 0.1 M (circles), 0.3 M (squares), 0.5 M (upward triangles) and 1 M (downward triangles) [25]. The inset shows the *X*^2^ vs. *t* plot.

The gel growth behaviors from the interface of polymer solutions with various types of gelator solutions have been observed. In Figure 1 we show an illustration of one of the simplest cases, the one-dimensional growth of a gel in a polymer solution cell in contact with a gelator solution bath (left-hand side of the cell). This model was proposed for the analysis of gelation dynamics of chitosan solution induced by a change of pH. Figure 2 shows a typical observed time course of the gel layer thickness *X*(*t*) induced at the interface of chitosan solution with NaOH solution [25]. Chitosan solution is soluble at a low pH and forms a gel at a neutral pH by hydrogen bonding. Under the geometry shown in Figure 1, the part of the chitosan solution where the pH changes from a low pH to a neutral pH is transformed to a gel. The initial process appears to be expressed by square-root behavior $X\sim \sqrt{t}$, as shown in the inset of Figure 2, suggesting diffusion-limited dynamics. Here we modify the model shown in Figure 1 and generalize it by including various cases in which A and B have different roles other than those of NaOH solution and chitosan solution, respectively. For example, the outflow of B may or may not be involved in gelation and gelation can occur with or without the consumption of A in the generalized model. Similar initial behaviors have, however, been observed for different types of gelator solutions under various geometries, whereas the late-stage behaviors were different from each other [28,29,30]. 

### 2.2. Moving Boundary Picture for Gelation Dynamics

The MB picture proposed by Yamamoto et al. [28,29] describes the gelation dynamics in terms of the sol-gel interface motion caused by the inflow of the gelator. The idea of the MB picture will be briefly explained through the gelation dynamics of chitosan solution. The dynamics introduced in Section 2.1 is analyzed as follows. In the system, the chitosan solution is enclosed in an L (length) × W (width) × D (depth) rectangular-solid space as shown in Figure 1 and is immersed in NaOH solution. The chitosan solution is in contact with the NaOH solution at a W×D side surface (the contact surface) [30].

The motion of the boundary x=X(t) is derived from the following assumptions.

(A) The sodium ions flow into the sol part and the acetate ions flow out from the sol part to the NaOH solution through the gel layer. The neutralization caused by the flows instantly results in the cross-linking of the inner chitosan solution to produce a new gel layer. 

(B) The gel layer does not capture the inflow sodium-ions and the outflow acetate-ions by acting as a sink and the flows change so slowly that they can be considered to be in a steady state; all the gelators inflowing from the gelator solution arrive at the inner polymer solution through the gel layer to realize a steady state.

(C) The NaOH solution, gel and inner sol are in local thermodynamic equilibrium at the boundaries.

The concentrations of sodium ions in the NaOH solution, in the inner sol, in the gel layer, at the interface between the gel and the NaOH solution at the position x=x in the gel layer and at the interface between the gel and the inner sol in the gel layer are respectively denoted by $\rho_{s}$, $\rho_{0}$, ρ(x), $\rho_{s}{}^{\prime }$ and $\rho_{0}{}^{\prime }$. The concentrations of acetate ions at the corresponding positions are respectively denoted by $C_{s}$, $C_{0}$, C(x), $C_{s}{}^{\prime }$ and $C_{0}{}^{\prime }$. The chemical potentials of the sodium ion in the NaOH solution, in the inner sol and in the gel layer are respectively denoted by $\mu_{NaOH}^{s}\left(\rho_{s}\right)$, $\mu_{NaOH}^{0}\left(\rho_{0}\right)$ and $\mu_{NaOH}\left(\rho \right)$ and those of the acetate ion are denoted by $\mu_{Ac}^{s}\left(C_{s}\right)$, $\mu_{Ac}^{0}\left(C_{0}\right)$ and $\mu_{Ac}\left(C\right)$. 

The flux of the inflow sodium-ion in the gel layer is expressed as

(1) $${\vec{j}}_{NaOH}\left(x\right)=j_{NaOH}\left(x\right){\vec{e}}_{x}$$

where $j_{NaOH}$ is the sodium ion flux density and is expressed in terms of the concentration ρ(x) and velocity $v_{NaOH}\left(x\right)$ of the sodium ion as $j_{NaOH}\left(x\right)=\rho \left(x\right)v_{NaOH}\left(x\right)$. The unit vector along the x-axis is denoted by ${\vec{e}}_{x}$. According to Fick’s law, the velocity of the sodium ion at x is given by

(2) $$v_{NaOH}\left(x\right)=-k_{NaOH}\frac{\partial \mu_{NaOH}\left(\rho \left(x\right)\right)}{\partial x}$$

where $k_{NaOH}$ is the mobility of the sodium ion. Therefore, we have

(3) $$j_{NaOH}\left(x\right)=-k_{NaOH}\rho \left(x\right)\frac{\partial \mu_{NaOH}\left(\rho \left(x\right)\right)}{\partial x}$$

In a similar way, we have the flux of the outflow acetate-ion as

(4) $${\vec{j}}_{Ac}\left(x\right)=-j_{Ac}\left(x\right){\vec{e}}_{x}$$

with

(5) $$j_{Ac}\left(x\right)=k_{Ac}C\left(x\right)\frac{\partial \mu_{Ac}\left(C\left(x\right)\right)}{\partial x}$$

In the above, $k_{Ac}$ is the mobility of the acetate-ion.

Let a new gel layer thickness dX be produced during period dt by neutralization caused by the sodium ion inflow. Assumption (A) gives the following relationship:

(6) $$\frac{1}{\rho_{G}\left(C_{0}\left(t\right)\right)}j_{NaoH}\left(X\right)WDdt=WDdX$$

where $\rho_{G}\left(C_{0}\right)$ is the number of sodium ions required to neutralize a unit volume of the inner chitosan solution with acetate ion concentration $C_{0}$ and is reasonably assumed to be proportional to $C_{0}$,

(7) $$\rho_{G}\left(C_{0}\right)=\alpha C_{0}$$

where α is a positive constant. Note that $C_{0}$ is a function of the immersion time t. Hence, we have the time development equation for the gel thickness:

(8) $$\frac{dX}{dt}=\frac{1}{\alpha C_{0}\left(t\right)}j_{NaoH}\left(X\left(t\right)\right)$$

Assumption (B) requires the following relationships:

(9) $$\mathrm{div}{\vec{j}}_{NaOH}=\frac{\partial j_{NaOH}}{\partial x}=-k_{NaOH}\frac{\partial }{\partial x}\left[\rho \left(x\right)\frac{\partial \mu_{NaOH}\left(\rho \left(x\right)\right)}{\partial x}\right]=0$$

(10) $$\mathrm{div}{\vec{j}}_{Ac}=-\frac{\partial j_{Ac}}{\partial x}=-k_{Ac}\frac{\partial }{\partial x}\left[C\left(x\right)\frac{\partial \mu_{Ac}\left(C\left(x\right)\right)}{\partial x}\right]=0$$

Integrating the differential Equations (9) and (10), we have the steady-state flows of sodium ions and acetate ions:

(11) $$j_{NaOH}=k_{NaOH}\frac{g_{NaOH}\left(\rho_{s}^{\prime }\right)-g_{NaOH}\left(\rho_{0}^{\prime }\right)}{X}$$

(12) $$j_{Ac}=-k_{Ac}\frac{g_{Ac}\left(C_{s}{}^{\prime }\right)-g_{Ac}\left(C_{0}{}^{\prime }\right)}{X}$$

where $g_{NaOH}$ and $g_{Ac}$ are the pressures of sodium ions and acetate ions respectively given by

(13) $$g_{NaOH}\left(\rho \right)=\mu_{NaOH}\left(\rho \right)\rho -f_{NaOH}\left(\rho \right)$$

(14) $$g_{Ac}\left(C\right)=\mu_{Ac}\left(C\right)C-f_{Ac}\left(C\right)$$

Here, the free energies $f_{NaOH}$ and $f_{Ac}$ per unit volume satisfy the relationships $\mu_{NaOH}\left(\rho \right)=\partial f_{NaOH}\left(\rho \right)/\partial \rho$ and $\mu_{Ac}\left(\rho \right)=\partial f_{Ac}\left(\rho \right)/\partial \rho$.

Assumption (C) gives the chemical potential balance

(15) $$\{\begin{array}{c} \mu_{NaOH}\left(\rho_{s}{}^{\prime }\right)=\mu_{NaOH}^{s}\left(\rho_{s}\right)\\ \mu_{NaOH}\left(\rho_{0}{}^{\prime }\right)=\mu_{NaOH}^{0}\left(\rho_{0}\right)\\ \mu_{Ac}\left(C_{s}{}^{\prime }\right)=\mu_{Ac}^{s}\left(C_{s}\right)\\ \mu_{Ac}\left(C_{0}{}^{\prime }\right)=\mu_{Ac}^{0}\left(C_{0}\right) \end{array}$$

Since the concentration of acetic acid is not so large, the chemical potential balance can be rewritten by assuming continuity of the concentration, $C_{s}{}^{\prime }≅C_{s}$ and $C_{0}{}^{\prime }≅C_{0}$. In the immersion solution, the concentration of acetic acid is very small, $C_{s}≅0$, because the volume of the immersion solution is very large. Hence, $C_{s}{}^{\prime }≅C_{s}≅0$. Therefore, the flux of the acetate-ion flow is given by

(16) $$j_{Ac}=-k_{Ac}\frac{g_{Ac}\left(0\right)-g_{Ac}\left(C_{0}\right)}{X}$$

In the dilute limit of acetate ions in the gel, the pressure is expressed by $g_{Ac}\left(C\right)=k_{B}TC$ and the flux is given by

(17) $$j_{Ac}=\frac{\beta C_{0}}{X}$$

where $\beta =k_{Ac}k_{B}T$. The acetate ion concentration in the sol part $C_{0}$ decreases with increasing immersion time t since acetate ions flow out from the sol part and the time development of the acetate ion concentration is given by

(18) $$V\left(X\right)\frac{dC_{0}\left(t\right)}{dt}=-WDj_{Ac}$$

where V(X)=WD(L−X) is the volume of the sol part. Using Equations (15) and (16), we have

(19) $$\frac{dC_{0}\left(t\right)}{dt}=-\frac{\beta C_{0}\left(t\right)}{L-X\left(t\right)}$$

Treating the sodium ion flow in the same manner as the acetate ion flow, we have

(20) $$j_{NaOH}=\frac{\gamma \rho_{s}}{X}$$

where $\gamma =k_{NaOH}k_{B}T$. 

Solving the simultaneous equations, Equations (8), (19) and (20), with the initial condition $C_{0}\left(0\right)=C_{in}$ and X(0)=0 and introducing the scaled variables $\tilde{X}=X/L$ and $\tilde{t}=t/L^{2}$, we have the scaled equation

(21) $$\tilde{\zeta }\left(\tilde{X},\frac{\beta }{K_{in}}\right)=K_{in}\tilde{t}$$

with

(22) $$\tilde{\zeta }\left(\tilde{X},\frac{\beta }{K_{in}}\right)=\int_{0}^{\tilde{X}}\frac{\tilde{u}}{1-\frac{\beta }{K_{in}}\ln\left(1-\tilde{u}\right)}d\tilde{u}$$

and

(23) $$K_{in}=\frac{\gamma }{\alpha }\frac{\rho_{s}}{C_{in}}$$

From the expansion $\tilde{\zeta }\left(\tilde{X},\frac{\beta }{K_{in}}\right)=\frac{1}{2}{\tilde{X}}^{2}+O\left({\tilde{X}}^{3}\right)$ around $\tilde{X}=0$, we derive the initial-stage ($\tilde{X}\approx 0$) behavior as

(24) $$\frac{1}{2}{\tilde{X}}^{2}\left(\tilde{t}\right)=K_{in}\tilde{t}$$

This equation indicates square-root behavior in which the gel thickness increases proportionally to the square of the immersion time,

(25) $$X=\sqrt{2K_{in}t}$$

The square-root behavior is a characteristic feature of the diffusion-limited dynamics.

**Figure 3:**
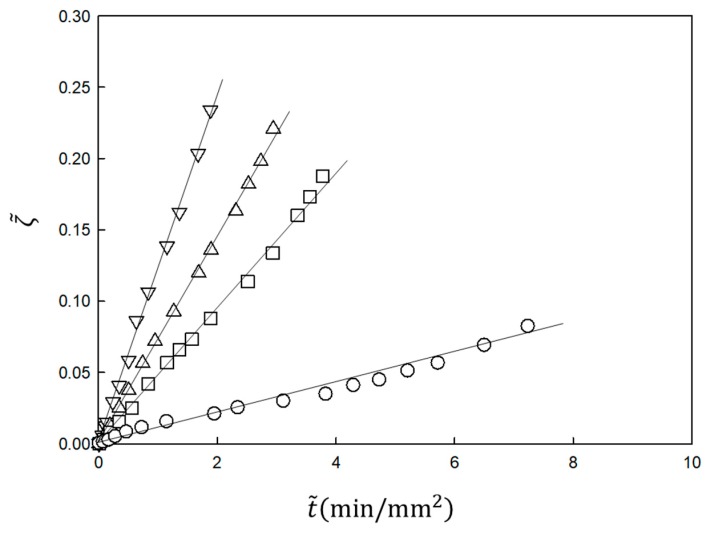
Time course of gel thickness obtained using the function ζ given by Equation (22) [30] for the data in Figure 2. ζ is proportional to $\tilde{t}$, as predicted by Equation (21).

The experimental results shown in Figure 2 are analyzed using Equations (21) and (22). In the analysis, $K_{in}$ and β are the fitting parameters. The results are plotted according to the equations in Figure 3. The slope *K_in_* is proportional to the NaOH concentration in the immersion solution, inversely proportional to the acetic acid concentration in the chitosan solution and independent of the chitosan concentration, as predicted by Equation (23) [30] (not shown). Therefore, the time course of the gel thickness is fully explained by the MB picture. The observed gelation behavior is expressed by the scaled Equation (21) with system-dependent coefficients $K_{in}$ and β. Thus, we can obtain information on the kinetic coefficients *γ* and *k_Ac_* of the system from the fitting parameters $K_{in}$ and β by comparing the experimental data with the theoretical equation.

### 2.3. Classification of Gelation Dynamics

In the gelation of chitosan solution, there are three types of characters having different roles. The first character is chitosan molecules, which are the element polymers constituting the gel. The second character is sodium ions, which are the gelator and are consumed to produce a gel layer. The third character is acetate ions, which are the gelation inhibitor.

Focusing on the gelator and the gelation inhibitor, we classify the gelation system into several types depending on the gelation mechanism. The simplest case is Case 1, in which cross-linking occurs simply by the inflow of cross-linkers as gelators; the gelation inhibitors are absent and the gelators are consumed to produce a gel layer. In Figure 1, the gelator A is involved in gelation whereas B is not involved in gelation. An example of Case 1 is a system where sodium alginate aqueous solution is in contact with calcium chloride aqueous solution (alginate/Ca^2+^ system) [4,5,6,7,8,9,10,20], in which calcium ions are the gelators. In Case 2, gelators flow into the element polymer solution and the gelation inhibitors flow out. Examples of Case 2 are polymers such as chitosan and collagen that undergo gelation via the formation of hydrogen bonds induced by a change in pH resulting from contact with a high pH solution. When chitosan or collagen in acetic acid solution with a low pH comes in contact with an aqueous solution of NaOH with a high or medium pH, anisotropic gels are prepared [15,30]. The gelator may be a catalyst, as in Case 3, making it necessary to consider the repeated use of the catalyst. In Case 3, although the inhibitors are absent, as in Case 1, the gelators are *not* consumed in the gelation. An example of Case 3 is the system where gelatin aqueous solution is in contact with transglutaminase aqueous solution [34]. 

Note that transient viscoelastic change occurs without any reactions when both solutes in liquid phases have very different diffusion constants [35]. We define this case as Case 4. In Case 4, the inhibitors are absent, as in Case 1. However, the gelator makes *no* links between the polymers. An example of Case 4 is the system where a high-molecular-weight DNA aqueous solution sandwiched between a pair of cover glasses is immersed in a low-molecular-weight DNA aqueous solution. In this case, the initial inflow of low-molecular-weight DNA causes the high-molecular-weight DNA solution to transiently form an anisotropic gel-like substance owning to the excluded volume effect and then the high-molecular-weight DNA diffuses to the immersion solution, finally resulting in a homogeneous solution. The gelation dynamics of the above-mentioned typical diffusion-limited systems were derived in Section 2.2 and are summarized in Table 1. 

Case 5 is similar to the crystal growth from solutions at low supersaturation. Assume that the free energy of a polymer solution has a double minimum with different values. The lower and higher values correspond to gel phase and sol phase, respectively. If a gel region is nucleated by contact with a solid phase, then the polymer solution gels and the front line of the gel moves forward. In Case 5, neither gelators nor gelation inhibitors are present. The gelation is not diffusion-limited in this case. An example of Case 5 is the system where plasma is in contact with packed erythrocytes, which is discussed in detail in Section 4.2. The cases given above are not exhaustive but are those for which examples are reported [28,29,30,31,32,33,34,35].

## 3. Theoretical View of Heterogeneous Gelation from the Standpoint of Phase Transition Dynamics

Here we regard gelation as a phase transition and discuss the gelation dynamics in the context of the phase transition dynamics [36,37,38] for the polymer solution in contact with the gelator solution. Let us discuss the gelation system corresponding to Case 1 as the simplest case. The state of the polymer solution depends on the concentration ρ of gelators in the polymer solution. The state of the polymer solution is described in terms of the *order parameter* ψ, which expresses the degree of gelation; ψ>0 when the polymer solution is in the gel state and ψ=0 when it is in the sol state. Then, the *local free energy* of the polymer solution is a function of ρ and ψ,

(26) f=f(ρ,ψ)

Let us consider a one-dimensional gel growth system, as shown in Figure 1. The free-energy functional expressing the whole of the polymer solution is given by

(27) $$F\left(\left\{\rho \right\},\left\{\psi \right\}\right)=\int \left[\frac{1}{2}\kappa_{\rho }{\left(\frac{\partial \rho }{\partial x}\right)}^{2}+\frac{1}{2}\kappa_{\psi }{\left(\frac{\partial \psi }{\partial x}\right)}^{2}+f\left(\rho \left(x\right),\psi \left(x\right)\right)\right]dx$$

where the positive constants $\kappa_{\rho }$ and $\kappa_{\psi }$ express the increase in free energy with respect to the space inhomogeneity of the gelator concentration and the degree of gelation, respectively. Since the concentration of gelators is a conserved quantity, the time development equation for the gelator is given by

(28) $$\frac{\partial \rho \left(x,t\right)}{\partial t}=\mathrm{div}\left[\Gamma_{\rho }\nabla \frac{\delta F}{\delta \rho \left(x,t\right)}\right]$$

where t denotes the elapsed time and $\Gamma_{\rho }$ is the kinetic coefficient for ρ. Since ψ is an order parameter and is not a conserved quantity, the time development equation for ψ is given by

(29) $$\frac{\partial \psi \left(x,t\right)}{\partial t}=-\Gamma_{\psi }\frac{\delta F}{\delta \psi \left(x,t\right)}$$

where $\Gamma_{\psi }$ is the kinetic coefficient for ψ. The boundary conditions

(30) $$\mu_{G}\left(\rho \left(0,t\right),\psi \left(0,t\right)\right)=\mu_{S}$$

is imposed, where $\mu_{G}\left(\rho \left(0,t\right),\psi \left(0,t\right)\right)$ and $\mu_{S}$ are, respectively, the chemical potential of the gelator in the polymer solution at the liquid-liquid contact interface and that in the gelator solution. In terms of F, $\mu_{G}$ is generally given by

(31) $$\mu_{G}\left(\rho \left(x,t\right),\psi \left(x,t\right)\right)=\frac{\delta F}{\delta \rho \left(x,t\right)}$$

One of the most characteristic features of the heterogeneous gelation process is that the boundary between the sol phase and gel phase is macroscopically clear. Thus, the gel growth dynamics can be visualized by the MB picture. The dynamics shown in Table 1 was derived by means of the MB picture.

Assumptions (A)–(C) in Section 2.2 for the gelation of chitosan solution (Case 2) is modified for Case 1 as follows [28] since the outflow ions are not involved in gelation in Case 1.

(A’) All the inflow gelators arriving at the inner polymer solution instantly cross-link the polymers to produce a new gel layer. 

(B’) The gel layer does not capture the inflow gelators by acting as a sink and the gelator flow changes so slowly that it can be considered to be in a steady state; all the gelators inflowing from the gelator solution arrive at the inner polymer solution through the gel layer to realize a steady state.

(C’) The gelator solution, gel and inner polymer solution are in local thermodynamic equilibrium at the boundary.

According to the above three assumptions, in terms of the concentration ρ(x) and chemical potential $\mu_{G}(\rho \left(x\right)$) of the gelator in the gel layer, the gelator flow density j(x) is given by

(32) $$j\left(x\right)=-k\rho \left(x\right)\frac{\partial \mu_{G}\left(\rho \left(x\right)\right)}{\partial x}$$

where k is the mobility of the gelator and we modify Equations (8), (19) and (20) governing the time development equations for the sol-gel boundary X(t) as [22]

(33) $$\{\begin{array}{c} \frac{dX\left(t\right)}{dt}=\frac{j\left(X\left(t\right)\right)}{\rho_{G}}\\ j\left(X\right)=kk_{B}T\frac{\rho_{s}}{X} \end{array}$$

where $\rho_{G}$ is the number of gelators required to produce a unit volume of gel. Note that $\rho_{G}$ is constant and the equation corresponding to Equation (18) is absent in Case 1 since gelation inhibitors are absent. 

Using the initial condition X(0)=0, we obtain the solution of Equation (33) as

(34) $$X\left(t\right)=\sqrt{2Kt}$$

where $K=kk_{B}T$. For Case 1, the square root behavior occurs during the entire period, although the behavior only occurs during the initial stage for Case 2.

Let us compare the above results with the phase transition dynamics given by Equations (28) and (29). The MB picture does not refer to the time development of the degree of gelation. Assumption (A’), however, requires that the time development of ψ is very rapid and an equilibrium value that depends on the gelator density ρ is quickly achieved. Thus, the degree of gelation ψ is expressed as

(35) $$\psi \left(x,t\right)\approx \psi_{0}\Theta \left(X\left(t\right)-x\right)$$

where for simplicity we let the “level function” Θ(x) be given by the step function defined by

(36) $$\Theta \left(x\right)=\{\begin{array}{cc} 0 & x<0\\ 1 & x>0 \end{array}$$

and $\psi_{0}$ is a positive constant satisfying the equation

(37) $$\frac{\partial f\left(\rho_{G},\psi_{0}\right)}{\partial \psi_{0}}=0$$

The gelator concentration in the MB picture indicates the concentration δρ(x) of the gelator dissolved in the solvent, which is given by

(38) $$\delta \rho \left(x\right)=\rho \left(x\right)-\rho_{G}$$

Therefore, in the context of phase transition dynamics, Equation (32) should be rewritten as

(39) $$j\left(x\right)=-k\delta \rho \left(x\right)\frac{\partial \mu_{G}\left(\rho \left(x\right)\right)}{\partial x}$$

Equation (39) requires the relationship

(40) $$\Gamma_{\rho }=\Gamma_{\rho }\left(\rho ,\psi_{0}\right)=k\delta \rho$$

for the kinetic coefficient $\Gamma_{\rho }=\Gamma_{\rho }\left(\rho ,\psi \right)$. Assumption (B’) requires that the gelator flow can be regarded as a steady flow. The time dependence of the gelator concentration ρ is very weak and appears only through the time dependence of the boundary X(t); the boundary condition, that is, Assumption (C’), determines the weak time dependence. Hence, the gelator concentration is given by

(41) $$\rho \left(x,t\right)\approx (\rho_{G}+\delta \rho \left(x;X\left(t\right)\right))\Theta \left(X\left(t\right)-x\right)$$

The chemical potential $\mu_{G}\left(x\right)$ in Equations (32) and (39) is given by

(42) $$\mu_{G}\left(x\right)=\frac{\partial f\left(\rho_{G}+\delta \rho \left(x\right),\psi_{0}\right)}{\partial \delta \rho \left(x\right)}$$

To effectively use the description of the phase transition dynamics given by Equations (28) and (29), it is necessary to clarify the function forms of f(ρ,ψ) and $\Gamma_{\rho }$ and the value of the kinetic constant $\Gamma_{\psi }$ statistical-mechanically and/or experimentally. This requirement will be discussed as a future issue. Here, we discuss a problem independent of the details of f(ρ,ψ) and $\Gamma_{\rho }$. Equation (33), expressing the gelation dynamics, is valid when the dynamics of ψ(x,t) is very fast compared with the gelator diffusion. What kind of dynamics is obtained in the opposite case, in which the dynamics of ψ(x,t) is very slow? The answer is obtained from the common phase transition dynamics. In this case, Equations (28) and (29) are rewritten as

(43) $$\frac{\partial f\left(\rho_{eq},0\right)}{\partial \rho_{eq}}=0$$

(44) $$\frac{\partial \psi \left(x,t\right)}{\partial t}=-\Gamma_{\psi }\frac{\delta F_{\psi }}{\delta \psi \left(x,t\right)}$$

with

(45) $$F_{\psi }=\int \left[\frac{1}{2}\kappa_{\psi }{\left(\frac{\partial \psi }{\partial x}\right)}^{2}+f\left(\rho_{eq},\psi \right)\right]dx$$

The boundary condition is given by

(46) $$\{\begin{array}{c} \psi \left(0,t\right)=\psi_{eq}\\ \underset{x\to \infty }{\lim}\psi \left(x,t\right)=0 \end{array}$$

where the equilibrium value of the degree of gelation $\psi_{eq}$ is obtained from the equation

(47) $$\frac{\partial f\left(\rho_{eq},\psi_{eq}\right)}{\partial \psi_{eq}}=0$$

The dynamics described by Equation (44) is limited by the free energy and is regarded as the relaxation from an unstable state to a stable state minimizing the free energy.

When $f\left(\rho_{eq},\psi \right)$ is a double-well-type function (both the sol phase (ψ=0) and the gel phase ($\psi =\psi_{eq}$) locally minimize the local free energy $f\left(\rho_{eq},\psi \right)$) and the constant $\kappa_{\psi }$ is small, the solution of Equation (44) is approximately [37,39]

(48) $$\psi \left(x,t\right)\approx \psi_{eq}\Theta \left(X\left(t\right)-x\right)$$

In this case, the gelation dynamics is also visualized by the motion of the sol-gel boundary. Therefore, to pay attention to the motion of the sol-gel boundary is valid. We could call the moving boundary picture the analysis method in which we pay attention to the sol-gel boundary motion even in the case of the energy-limited dynamics. The time dependence of X(t), however, differs from the square-root behavior. The dynamics is expected to effectively visualize the gelation dynamics for Case 5. 

Finally, we discuss how the anisotropy is taken into account. The presence of a gelator concentration gradient ∂ρ(x)/∂x during gelation is one of the characteristics of heterogeneous gelation. Therefore, we assume that the local free energy depends on the concentration gradient and introduce the order parameter ϕ expressing the degree of anisotropy of the polymer solution, where ϕ>0 when the polymer solution is anisotropic and ϕ=0 when it is isotropic. The local free energy is given by

(49) $$f=f\left(\rho ,\frac{\partial \rho }{\partial x},\psi ,\phi \right)$$

The free-energy functional is given by

(50) $$F\left(\rho ,\frac{\partial \rho }{\partial x},\psi ,\phi \right)=\int \left[\frac{1}{2}\kappa_{\rho }{\left(\frac{\partial \rho }{\partial x}\right)}^{2}+\frac{1}{2}\kappa_{\psi }{\left(\frac{\partial \psi }{\partial x}\right)}^{2}+\frac{1}{2}\kappa_{\phi }{\left(\frac{\partial \phi }{\partial x}\right)}^{2}+f\left(\rho ,\frac{\partial \rho }{\partial x},\psi ,\phi \right)\right]dx$$

where the positive constant $\kappa_{\phi }$ expresses the increase in free energy with respect to the space inhomogeneity of the anisotropy. In addition to Equations (28) and (29), we introduce the time development equation for ϕ as

(51) $$\frac{\partial \phi \left(x,t\right)}{\partial t}=-\Gamma_{\phi }\frac{\delta F}{\delta \phi \left(x,t\right)}$$

where $\Gamma_{\phi }$ is the kinetic coefficient for ϕ.

Let us discuss the diffusion-limited gelation dynamics with the anisotropy expressed by the MB picture. The gelation dynamics of curdlan solution [28] is in this category. In this case, the time development of the anisotropy is expressed as

(52) $$\phi \left(x,t\right)\approx \phi_{0}\Theta \left(X\left(t\right)-x\right)$$

where

(53) $$\frac{\partial f\left(\rho_{G},\frac{\partial \delta \rho \left(X\right)}{\partial X},0,\phi_{0}\right)}{\partial \phi_{0}}=0.$$

Equation (53) requires the local free energy to reach a local minimum at a finite value of the degree of anisotropy. Note that global minimization of the local free energy at a finite ϕ is not required. At a concentration gradient $\left|\frac{\partial \delta \rho \left(X\right)}{\partial X}\right|$ larger than a threshold value, the local free energy is expected to have such a local minimum. The increase in the degree of gelation roughly coincides with the increase in anisotropy. The kinetic coefficient $\Gamma_{\phi }$ rapidly drops to zero with increasing ψ since the gelation significantly interferes with the polymer motion in the polymer solution. Thus, the degree of anisotropy is fixed at a finite value $\phi_{0}$.

## 4. Application of Scaled Gelation Dynamics to Analysis of Blood Coagulation

**Figure 4:**
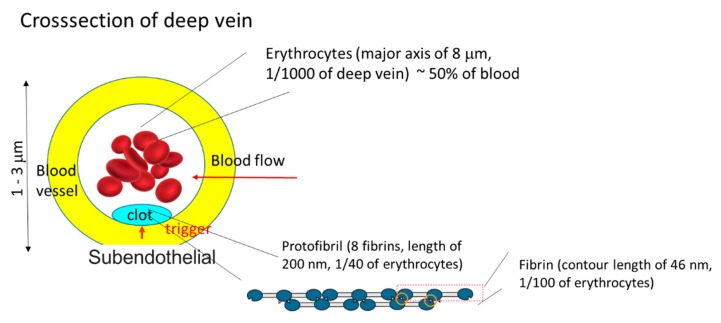
Illustration of blood coagulation at blood vessel wall.

One of the most interesting applications of the analysis of heterogeneous gelation dynamics is blood coagulation (gelation). Blood consists of about 45% blood corpuscles, which are mainly erythrocytes and the remaining liquid component, plasma. Fibrinogens, which comprise 7% of blood protein, are the main component of coagulants (gels). Blood coagulation is triggered by the contact of plasma with coagulant factors at cell surfaces or blood vessel walls, as illustrated in Figure 4. Therefore, the main process of macroscopic blood coagulation is the gelation of plasma induced by the contact of plasma and coagulant factors at a cell surface. With a sufficient amount of calcium ions, after complex cascade reactions, the key protein thrombins are activated and fibrinogens are hydrolyzed to form fibrins and then protofibrils, which are the building blocks of the coagulant. The biochemical reaction cascade in blood coagulation has been established by considering a number of simplified homogeneous systems [40]. However, the research on the dynamic aspects of blood coagulation by considering heterogeneous systems, such as a thrombin solution/plasma contact system, began relatively recently [41,42]. Determination of the kinetic coefficients of blood coagulation should help provide the missing link between the biochemical properties and disorders in blood coagulation. The kinetic coefficients can potentially be used as direct indicators of blood coagulability in diagnosis. 

It is very difficult to completely replace an in vivo system by an in vitro system for blood testing, because when we draw blood from a body we must add some anticoagulants such as citrates or heparin to the blood. Therefore, it is important to study key systems in which part of the blood coagulation process can be reproduced to obtain useful information on blood. Here we discuss two systems of interest, citrated plasma in contact with calcium chloride aqueous solution (plasma/Ca^+^) and citrated plasma in contact with packed erythrocytes (plasma/packed erythrocytes). 

### 4.1. Analysis of Citrated Plasma/Ca^+^ Contact System 

In the clinical test to assess blood coagulability, a trigger protein or trigger lipid and calcium ions are mixed with citrated plasma and the clotting time is measured and compared with the standard value. Let us consider heterogeneous gelation as a control clinical testing system; this system consists of citrated plasma in contact with a calcium ion solution separated by a dialysis membrane, as illustrated in Figure 1. There is an inflow of calcium ions and an outflow of citrate ions through the dialysis membrane. When the calcium ion concentration in the plasma exceeds the threshold, gelation occurs. If we replace citrate ions, calcium ions and fibrinogen by acetate ions, sodium ions and chitosan, respectively, this system corresponds to Case 2 [43]. Therefore, the measurement of the gel layer thickness *X* as a function of time after the liquid-liquid contact *t* can be compared with the theoretical equation, Equation (T2-1) in Table 1, of

(54) $$\tilde{\zeta }\left(\tilde{X},\frac{\beta }{K_{in}}\right)=\int_{0}^{\tilde{X}}\frac{\tilde{u}}{1-\frac{\beta }{K_{in}}\ln\left(1-\tilde{u}\right)}d\tilde{u}=K_{in}\tilde{t}$$

where

(55) $$\tilde{X}=X/L,\;\tilde{t}=t/L^{2}$$

and *L* is the effective length of the cell. Since the blood coagulation dynamics is expressed by the scaled form of Equation (54), the analytical results based on the equation do not depend on the experimental tools. This is an advantage of this method of extracting valuable information on blood.

**Figure 5:**
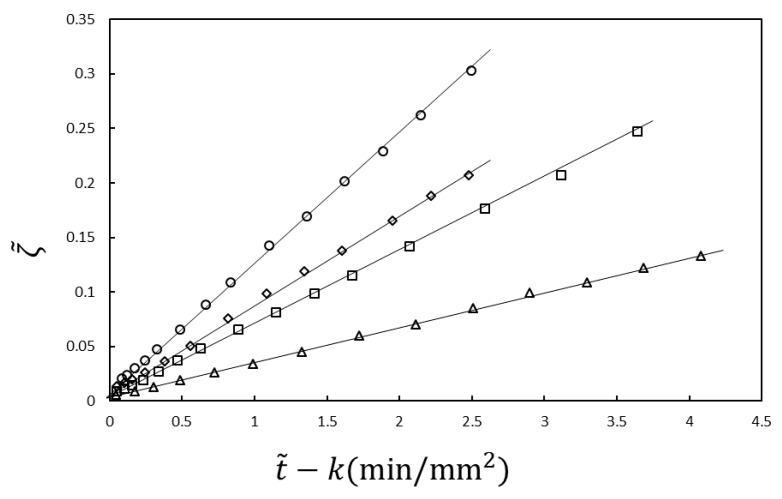
Gelation dynamics expressed by Equation (54) for horse plasma in contact with calcium chloride with concentrations of 10 mM (△), 30 mM (□), 50 mM (◊) and 100 mM (○).

Recently the entire time course of the gel thickness was compared with the theory for bovine blood and was found to be well expressed by Equation (54) [43]. However, since the blood coagulation behavior of animals of the even-hoof class, such as cattle, is known to be considerably different from that of other animals such as human, swine and horses [44], in this paper we performed the corresponding experiment using blood from a horse, one of the animals of the odd-hoof class. The time course was well expressed by Equation (54) as shown in Figure 5. The scaled time lag *k* used for fitting was close to the clotting time, which was the time required for macroscopic gelation by mixing (homogeneous gelation), used in clinical testing. Therefore, the analysis is also applicable for the blood of animals other than even-hoof animals. Note that the observed data could only be fit to Case 2 among the five cases. Calcium ions are known to play various roles in blood. They bind plasma proteins that are both involved and not involved in coagulation, each with a different binding constant. For example, fibrinogen has several strong and weak binding sites with calcium ions. Some enzymes are only activated in the presence of a sufficient amount of calcium ions. Thus, blood coagulation is generally complex. The present system is regarded as a simplified one to relate the observed gelation dynamics to Case 2. Here, we further expand the expressions for parameters *β* and *K_in_*. From the definition, we have

(56) $$\beta =\frac{\alpha \beta_{0}C_{in}}{\rho_{G}^{0}+\alpha C_{in}}$$

(57) $$\beta_{0}=k_{CIT}k_{B}T$$

(58) $$K_{in}=\frac{\gamma \rho_{s}}{\rho_{G}^{0}+\alpha C_{in}}$$

*γ* = *k_Ca_k_B_T* (59)

where *C_in_* is the initial concentration of citrate in the plasma, *ρ_s_* is the initial concentration of calcium ions in the calcium chloride aqueous solution, *γ* and *β*_0_ are the diffusion constants of calcium ions and citrate ions in the gel, respectively and $\rho_{G}^{0}$ is the calcium ion concentration required for the gelation of a unit volume of plasma. From Equations (56)–(59), we have

(60a) $$C_{in}=-\rho_{G}{}^{0}+\gamma \frac{\rho_{s}{}^{0}}{K_{in}}$$

and

(60b) $$C_{in}=-\rho_{G}{}^{0}+\beta_{0}\frac{C_{in}}{\beta }$$

Therefore, by measuring the citrate concentration dependence of the parameters *β* and *K_in_*, we can determine the three physically defined quantities *γ*, *β*_0_ and *ρ*_G_^0^ from the plot using Equations (60a) and (60b). According to preliminary experiments, this analysis is also valid for human blood and the kinetic coefficients depends on subjects. Therefore, these parameters could be used for diagnosis as new indicators reflecting blood coagulability.

### 4.2. Analysis of Plasma/Packed Erythrocyte Contact System

**Figure 6:**
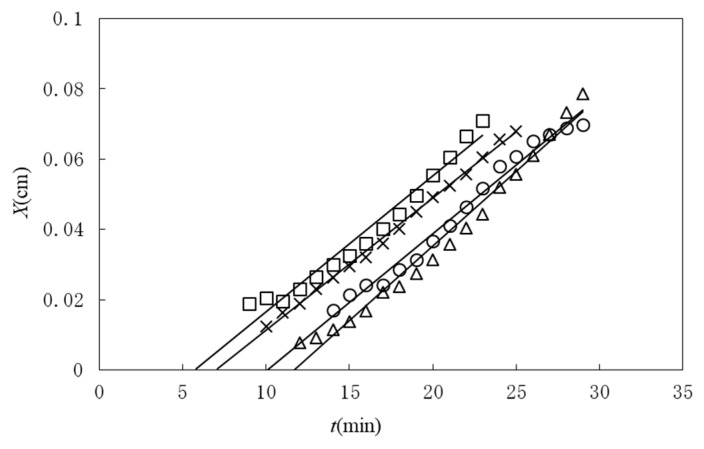
Time course of gel layer thickness observed on packed swine erythrocytes in contact with plasma with calcium chloride concentrations of 1.63 mM (○), 2.50 mM (△), 4.59 mM (□) and 6.68 mM (×).

**Figure 7:**
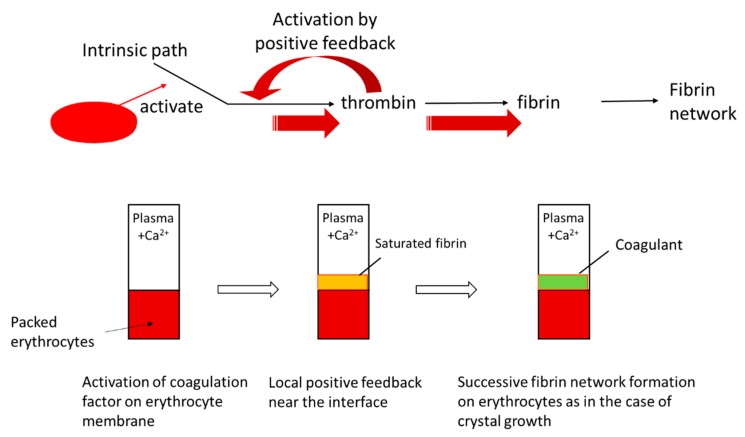
Scheme of coagulation induced by contact of plasma with packed erythrocytes.

Until recently, erythrocytes were regarded as being unrelated to blood coagulation. However, in the past few decades, several pieces of indirect evidence that erythrocytes have an active function in thrombosis and hemostasis have been found [45]. In particular, in the case of blood flow stagnation, such as in deep vein thrombosis, also called economy-class syndrome, the erythrocyte surface is suspected to be one of the factors that trigger blood coagulation [46]. Deep vein thrombosis is a blood clot that develops within a deep vein in the body, usually in the leg. When part of the blood clot breaks off, enters the bloodstream and blocks one of the blood vessels in the lungs, it leads to complications such as pulmonary embolism [47]. It has been assumed that protein C or protein S, both of which inhibit the coagulation cascade in the normal state, is inactivated, resulting in blood coagulation [48]. Kaibara et al. proposed a new pathway involving the activation of an intrinsic pathway in the coagulation cascade by the protein on the erythrocyte membrane, erythroelastase [46]. Although few continuous studies have been carried out on this pathway, the system involving the contact of erythrocytes and plasma seems to be appropriate for studying whether or not this scheme is consistent with or directly related to deep vein thrombosis. Because of the deformability of erythrocytes, we can easily prepare packed erythrocytes with a flat surface by centrifugation. In this study, we performed a study on gelation dynamics induced in swine plasma in contact with packed erythrocytes. Figure 6 shows the time course of the thickness of a gel grown from a packed erythrocyte surface. After a time lag, the gel thickness increased proportionally to time. The slope was roughly constant, irrespective of the calcium concentration, which means that the gelation behavior is the same above a threshold calcium concentration after gelation is initiated. According to the mechanism proposed by Kaibara et al., a coagulation protein called factor IX is activated on the surface of erythrocytes [46]. Then the coagulation cascade should be successively activated and positive feedback may yield a locally activated space with saturated fibrin molecules similarly to in vivo [49]. The fibrin molecules form a network from the surface of the packed erythrocytes, similarly to in the case of crystal growth, as illustrated in Figure 7. This situation is similar to Case 5. Choosing a quartic function of ψ in the function $f\left(\rho_{eq},\psi \right)$ and the boundary condition Equation (46), we obtain the solution of Equation (48) according to Chan [50]. The position of the sol-gel interface is expressed by the linear function of time

(61) $$X\left(t\right)=v\left(t-t_{0}\right)$$

where $t_{0}$ is the lag time required so that the boundary condition is satisfied and v is a positive constant depending on the free-energy difference $f\left(\rho_{eq},\psi_{eq}\right)-f\left(\rho_{eq},0\right)$. Therefore, the observed linear increase in *X* with time *t* shown in Figure 6 is explained by Equation (61) and the proportionality coefficient is related to the free-energy difference between the initial state and the equilibrium state. On the other hand, such a gel layer was hardly observed on hardened erythrocytes modified by glutaraldehyde whose plasma membrane was inactivated. Thus, the experimental results are consistent with the mechanism proposed by Kaibara et al. [46]. The rate of increase in the gel layer thickness provides information on the coagulability of the examined blood through the proportionality coefficient *v*. According to a preliminary experiment, this approach is valid for human blood. The kinetic coefficient determined by the observed heterogeneous gelation dynamics may be related to the coagulability of the blood of subjects and can potentially be used as an indicator of deep vein thrombosis. 

## 5. Conclusions

The gelation dynamics induced by the contact of polymer and gelator solutions was classified into several types and expressed by a system-independent scaling equation and system-dependent coefficients. From the viewpoint of the phase transition, it was clarified that the time taken for the development of the gelation layer depends on whether the dynamics of the order parameter expressing the gelation of the polymer solution is fast or slow compared with the diffusion of the gelators. As a straightforward application of one type of gelation dynamics (Case 2), in vitro blood coagulation was analyzed to find kinetic coefficients characterizing the dynamics. New information on blood from human subjects can be extracted by the analysis of gel growth behavior. Heterogeneous gelation is involved in many industrial processes such as microencapsulation by an insolubilization reaction to prepare artificial eggs and drug delivery carriers. Information on the extent of the reaction is important for controlling the retention and release capability of the core substance. For spherical entities, however, it is difficult to trace the gelation dynamics. The theoretical analysis in Section 2 may also be applicable to these materials to enabling the gel layer thickness to be estimated at a desired time.

## 6. Materials and Method

To study the plasma/Ca^+^ contact system, horse blood aseptically collected in equal volumes of Alsever’s solution was purchased from Nippon Bio-test Laboratories Inc. The blood was centrifuged at 1600× *g* for 10 min at 25 °C to obtain platelet-free plasma (PFP). The plasma was poured in a cell (10 mm in height, 3 mm in width and 20 mm depth) made of poly(methylmethacrylate) from the bottom to the upper end (inlet). Then the inlet was sealed with a dialysis membrane (Sanko Jun-yaku Co., Ltd., Tokyo, Japan) to confine plasma proteins such as fibrinogen in the plasma. The cell was immersed in 40 mL of calcium chloride aqueous solution with various calcium chloride concentrations in the range of 10–100 mM. The motion of the boundary was observed and recorded with a digital camera at 25 °C. 

To study the plasma/packed RBC contact system, swine blood provided by Gunma Meat Wholesale Market Co. Ltd. was citrated with 3.2% sodium citrate in a polypropylene tube. The suspension was centrifuged at 1600× *g* for 10 min at 25 °C to separate the PFP and packed erythrocytes. Hardened erythrocytes were prepared by mixing 20% erythrocyte suspension with 1.6% glutaraldehyde aqueous solution with 40 times the amount of erythrocyte suspension and gently stirring it for 72 h. Then the precipitates were rinsed with MilliQ water five times and physiological saline solution twice. After placing 2 mL of the PFP with the desired concentration of calcium ions on 1 mL of packed erythrocytes in a collagen-coated polystyrene tube, we observed the growth of a turbid layer from the interface over time at 25 °C. The volume of the turbid layer was estimated by image analysis and the gel volume was measured directly; these two volumes were found to be equivalent. Therefore, the growth of the gel layer was traced by observing the thickness of the turbid layer. 

## Figures and Tables

**Table 1:** Types of gelation and time development equation.

Case #	Geometry *	Time Development of Gel Thickness	System-Dependent Parameters	Equation #	Ref.
1	One-dimensional	$x=\sqrt{2Kt}$	$K=kk_{B}T\frac{\rho_{S}}{\rho_{G}}$	T1-1	
1	Cylindrically symmetrical	$\tilde{y}\equiv \frac{1}{2}{\left(1-\tilde{x}\right)}^{2}\;\ln\left(1-\tilde{x}\right)-\frac{1}{4}{\tilde{x}}^{2}+\frac{1}{2}\tilde{x}=K\tilde{t}$	$K=kk_{B}T\frac{\rho_{S}}{\rho_{G}}$	T1-2	[26]
2	One-dimensional	$\tilde{\zeta }(\tilde{X},\frac{\beta }{K_{in}})\equiv \int_{0}^{\tilde{X}}\frac{\tilde{u}}{1-\frac{\beta }{K_{in}}\ln\left(1-\tilde{u}\right)}d\tilde{u}=K_{in}\tilde{t}$	$K_{in}=\frac{k_{NaOH}k_{B}T}{\alpha }\frac{\rho_{s}}{C_{in}},\;\beta =k_{Ac}k_{B}T$	T2-1	[25]
2	Cylindrically symmetrical	$\tilde{z}\left(\tilde{x};\frac{2\beta }{K_{in}}\right)\equiv \int_{0}^{\tilde{x}}\frac{\left(1-\tilde{u}\right)\ln\frac{1}{1-\tilde{u}}}{1-\frac{2\beta }{K_{in}}\ln\left(1-\tilde{u}\right)}d\tilde{u}=K_{in}\tilde{t}$	$K_{in}=\frac{k_{NaOH}k_{B}T}{\alpha }\frac{\rho_{s}}{C_{in}},\;\beta =k_{Ac}k_{B}T$	T2-2	[28]
3	One-dimensional	$x=\sqrt{2Kt}$	$K=2k_{ct}k_{B}T$	T-3	
4	One-dimensional	$x=\sqrt{2Kt}$ (initial process)	$K=kk_{B}T\frac{\rho_{S}}{\rho_{G}}$	T1-1	

* One dimensional geometry corresponds to Figure 1. Cylindrically symmetrical geometry refers the case such as dialysis of polymer solutions to gelator solutions. *x*: gel thickness; $\tilde{x}$: gel thickness scaled by the radius of cylinder or the effective length of the cell; *t*: time; $\tilde{t}$: time scaled by square of the radius of cylinder or of the effective length of the cell; $\rho_{G}$: the number of gelator required to produce a unit volume of gel; $\rho_{S}$: concentration of gelator or acetate ion; $C_{in}$: initial concentration of sodium ion; α: a positive numerical factor; k: mobility of gelator; $k_{NaOH}$: mobility of sodium ion; $k_{Ac}$: mobility of acetate ion; $k_{ct}:$ mobility of catalyst; $k_{B}$: Boltzmann constant; *T*: absolute temperature.
